# Theory of frequency response of mechanically driven cardiomyocytes

**DOI:** 10.1038/s41598-018-20307-2

**Published:** 2018-02-02

**Authors:** Ohad Cohen, Samuel A. Safran

**Affiliations:** Dept. Chemical and Biological Physics, Weizmann Institute of Science, Rehovot, IL 76100 Israel

## Abstract

We theoretically predict and compare with experiments, transitions from spontaneous beating to dynamical entrainment of cardiomyocytes induced by an oscillating, external mechanical probe. In accord with recent experiments, we predict the dynamical behavior as a function of the probe amplitude and frequency. The theory is based on a phenomenological model for a non-linear oscillator, motivated by acto-myosin contractility. The generic behavior is independent of the detailed, molecular origins of the dynamics and, consistent with experiment, we find three regimes: spontaneous beating with the natural frequency of the cell, entrained beating with the frequency of the probe, and a “bursting” regime where the two frequencies alternate in time. We quantitatively predict the properties of the “bursting” regime as a function of the amplitude and frequency of the probe. Furthermore, we examine the pacing process in the presence of weak noise and explain how this might relate to cardiomyocyte physiology.

## Introduction

Recent experiments, models and theories have demonstrated the importance of mechanics in tissue development^1,2^, wound healing^3,4^, cancer metastasis and invasion^5,6^, tissue remodeling^7^, nuclear organization^8,9^, as well as in other biological phenomena. Adhesions^10,11^ couple the cellular cytoskeleton to the extracellular matrix *in-vivo*, or to an underlying substrate *in-vitro*^12^ whose mechanical deformations can modulate various structural and biological processes within the cell^10,13–15^.

In addition to their mechanosensitivity, cells are also able to apply physical forces to their surroundings^11,16^. This is achieved by active contraction of sarcomeric^17^ or sarcomere-like^18,19^ units, comprising actin filaments and myosin motors within the cytoskeleton. In muscle cells and fibroblasts, the local contraction of many such units is translated to a global contraction of the entire cell, and is transmitted by the adhesions to the surrounding matrix. Static mechanical deformations decay with power laws^10^, which contribute to long-range mechanical interactions between cells^14,20,21^.

This paper focuses theoretically on how external mechanical forces applied to a beating cardiac cell can synchronize its frequency. The ability to stimulate mechanically a quiescent cardiomyocyte using a mechanical probe that deform the underlying substrate was first shown by Tang *et al*.^22^, and synchronization of spontaneously beating cardiomyocytes with a similar probe was recently demonstrated in the experiments by Nitsan *et al*.^23^.

It is a well-established fact that the beating of cardiac cells is regulated through calcium dynamics^24^. An increase in cytoplasmic calcium ions concentration activates ryanodine receptor (RyR) channels embedded in the sarcoplasmic reticulum (SR), which causes further release of calcium ions to the cytoplasm in a process known as “calcium induced calcium release” (CICR). Calcium ions then bind to troponin, which exposes myosin binding sites on actin filaments. Myosin motors bind to actin, and contraction of the muscle ensues^24,25^. In tissue, contacting cardiomyocytes can synchronize their contraction through exchange of ions via shared gap junction^26^. Interestingly, cardiomyocytes separated over large distances (up to 300 *μm* away) by fibroblast cells can still synchronize their spontaneous beating^27^.

In addition to ionic signaling, Nitsan *et al*.^23^ have shown that purely mechanical signals can control the beating of cardiomyocytes at distances of over 100 microns away. In their experiments, Nitsan *et al*. showed that nearby neonatal cardiac cells, seeded ~100 micrometers apart on an elastic gel, synchronize their beating phase and frequency even without direct contact. By introducing an inert probe that induced periodic elastic deformations in the substrate, the experiments showed that one can pace beating cardiac cells (i.e. - synchronize their beating with the deformation of the substrate) that are not in direct contact, and are relatively far from the probe. Complete synchronization is apparent for a range of frequency differences between the spontaneous and probe frequency. When the difference in frequencies becomes large enough, the cells display “bursting” behavior, where intermittent periods of synchronized contraction and quiescence are observed. Interestingly, the bursting regime is characterized by several beats at the frequency of the probe, followed by a quiescent interval. The overall duration of both of these is comparable to the inverse of the spontaneous beating frequency of the cell. The time required to pace the cell was on the order of ~15 min, and the cell maintained the new beating frequency for as long as ~1 hr after the probe was removed. These long time scales for initiating and sustaining synchronized contraction are in complete contrast to the very short time scales (~1 sec) that characterize initiation and loss of entrainment associated with electrical stimulation of calcium oscillations^23^. This might indicate that mechanical feedback changes the spontaneous frequency of the cell, and suggests the existence of relatively long-lived, biological adaptation.

In this paper, we predict and compare with experiment^23^ the dynamical states of a beating cardiomyocyte, using a generic, one dimensional, non-linear oscillator model. The biophysical origin of spontaneous beating in cells is of great interest, and the non-linear properties can be obtained from one of the following scenarios (see SI, section A for further discussion):

1. Non-linear response of the active contractile machinery that yields spontaneous contraction oscillations of actin-myosin bundles. This option was first explored by Jülicher and Prost^28,29^, and was recently applied to hair cells in the ear, where the effect of varying the amplitude of an oscillating signal (sound wave) on these cells was examined. It was shown that the non-linear mechanical response is crucial for the excitation of hair bundles due to specific tone frequencies^30–32^. In this scenario, although calcium ions may be involved, there is no feedback between those and mechanical oscillations. That is, the external force paces the contractile machinery while spontaneous calcium oscillations remain unmodulated. Since it is known that the Ca^2+^ concentration in cardiomyocytes oscillates and drives the sarcomere beating^24,25^, this mechanism may not provide a complete description of beating in heart cells.
2. Spontaneous oscillations of calcium concentration arising from the biochemistry of the sarcomeric reticulum (SR). The non-linearity comes from the activation of ryanodine receptors (RyR) on the SR by calcium ions in the cytosol^24,25^. In this scenario, external mechanical deformations are either coupled to the sarcolemma^33^, or directly to the SR^34^ through adhesion points. The applied tension then modulates the activity of RyR channels on the SR that releases more calcium from the SR to the cytosol, which further feeds back into the CICR cycle, effectively modifying acto-myosin contraction (for further details, see Sec. A of the SI). The contractile machinery is thus driven by calcium.
3. Coupling of the SR oscillations to the acto-myosin units. In this scenario, the external mechanical force couples to the contractile machinery via integrin adhesions of the costameres^34^. Here, pacing of the contractile machinery feeds back into the calcium cycle and modulates SR channels activity. Thus, pacing induces both contractility and calcium oscillations.

Note that irrespective of the molecular-level scenarios above, the outcome is spontaneous acto-myosin contraction and/or calcium concentration oscillations. The generic, coarse-grained dynamics of these are ultimately determined by the external pacing mechanical force. Since we focus on the dependence of the dynamics on the amplitude and frequency of probe, as investigated in the recent experiments^23^, we analyze the behavior of a generic, non-linear oscillator that might result from any of these three scenarios (see Sec. A of the SI). While our theory is applicable to the pacing of a beating cell by an external, mechanical probe, the experiments have shown^23^ that similar considerations are relevant to the mechanical pacing of one cell by a neighboring one. Additional experiments are required in order to ascertain which of the three microscopic scenarios is more relevant to the observed response to pacing.

We begin in Sec. 2 with a simple, analytical treatment of the deterministic dynamics that predicts spontaneous, bursting, and entrained beating (with the probe frequency) of paced cells. The theory predicts how these depend on the probe amplitude and frequency, in agreement with experiment^23^.

In Sec. 3 we analyze the time required for a cell to transition from spontaneous to entrained beating once the probe is applied, and its dependence on the probe amplitude. We account for the origin of the much longer time scale (minutes) required to entrain spontaneously beating cells by considering biological adaptation (which delays the response of the cell to the external signal).

In Sec. 4, we consider the interesting effects of small noise in destabilizing the phase of the non-linear oscillator^35^. We also discuss the role of the probe in reducing the effects of noise.

### Dynamics of beating cells subject to mechanical force

Cardiac cells seeded on a visco-elastic substrate usually adopt an elongated shape, where the majority of the contraction force is applied at the two opposite edges along the long axis of the cell^36^. These cells tend to beat spontaneously with roughly constant amplitude (strain of ~5% relative to the cellular length of ~60–100 *μm*^23,37^) and a frequency of ~0.5–2 Hz. In this section, we analyze the steady-state, deterministic dynamics of both spontaneously beating cells (with frequency *ω*_*c*_) as well as those of cells that are subject to an external force (an adjacent cell, or a probe that mimics the deformation of a cell). For a cell in an elastic medium, we have shown^38^ how the amplitude of the applied force depends on the distance to the cell and the orientation of the other cell or probe. When this force is applied at a frequency *ω*_*p*_ that is different from *ω*_*c*_, the cell dynamics is changed in a non-linear and non-additive manner.

The elongated shape of a beating cardiomyocyte and the observations made by Nitsan *et al*.^23^ that calcium oscillations and sarcomere contraction are approximately spatially uniform for all times, motivates us to focus on the deformations along the long axis of cell, and adopt a coarse grained, one-dimensional model for a single oscillating degree of freedom *χ*(*t*)^39^:

1 $$\ddot{\chi }(t)+\rho \dot{\chi }(t)+{\rm{\lambda }}\,\dot{\chi }{(t)}^{3}+{\omega }_{c}^{2}\,\chi (t)={f}_{ext}(t)$$

A pedagogical derivation of this equation, as well as a discussion of the biological origin of these terms in the context of the three scenarios above, is presented in Sec. A of the supplementary information (SI). The first term represents an acceleration which is not at all related to inertia (mass) since cells reside in aqueous solutions where dissipation is dominant and inertia is negligible^10,40^. Rather, this term comes from the dynamical, active process of binding and unbinding of myosin motors to actin filaments, and/or the dynamics of activating ion channels embedded in the SR. The second term in general, represents the feedback due to changes of *χ*(*t*) over time (rate or velocity). This feedback can be either negative (suppresses changes in *χ*(*t*) - e.g. friction) or positive (amplifies changes in *χ*(*t*) - e.g. energy input). Large increases in *χ*(*t*) are eventually suppressed by the higher order term in the rate/velocity with *λ* > 0. The term linear in *χ*(*t*) represents a restoring force that opposes changes in *χ*(*t*) from its steady-state value, defined to be zero. When the parameter *ρ* switches sign from positive to negative, the system undergoes a bifurcation, and displays spontaneous oscillations with frequency *ω*_*c*_ and a steady-state amplitude ${A}_{s}=\sqrt{4\rho /3\lambda {\omega }_{c}^{2}}$. *f*_*ext*_*(t)* is a time dependent, external pacing force. Note that both the frequency and amplitude *A*_*s*_ can be measured experimentally without knowing the values of, *ρ* and *λ*.

The three biophysical scenarios outlined above (and in the SI) each result in a different interpretation of the coarse-grained degree of freedom, *χ*(*t*). In scenario (1), *χ*(*t*) is the physical displacement of the acto-myosin contractile units (sarcomeres) coupled to the deformations induced by the probe. In (2), *χ*(*t*) is the ionic calcium concentration in the cytosol, and the external force applies tension to the SR/sarcolemma - affecting calcium release. In (3), *χ*(*t*) is the ionic concentration of calcium, coupled to the contractile machinery, which is, in turn, coupled to the external force. We elaborate on the three scenarios in Sec. A1 of the SI. In the following, we use the theory to predict generic properties of the oscillations, independent of the microscopic scenario.

Motivated by the experiments^23^ in which cells are paced by either a probe or other cells, we consider an applied force oscillating with a frequency *ω*_*p*_ that, in general, is not equal to the spontaneous beating frequency of the cell, *ω*_*c*_. We choose a simple form for the time dependent force ${f}_{ext}(t)={A}_{p}\,\cos ({\omega }_{p}t)$ where *A*_*p*_, is the amplitude of the force measured in the vicinity of the cell^38^.

**Figure 1:**
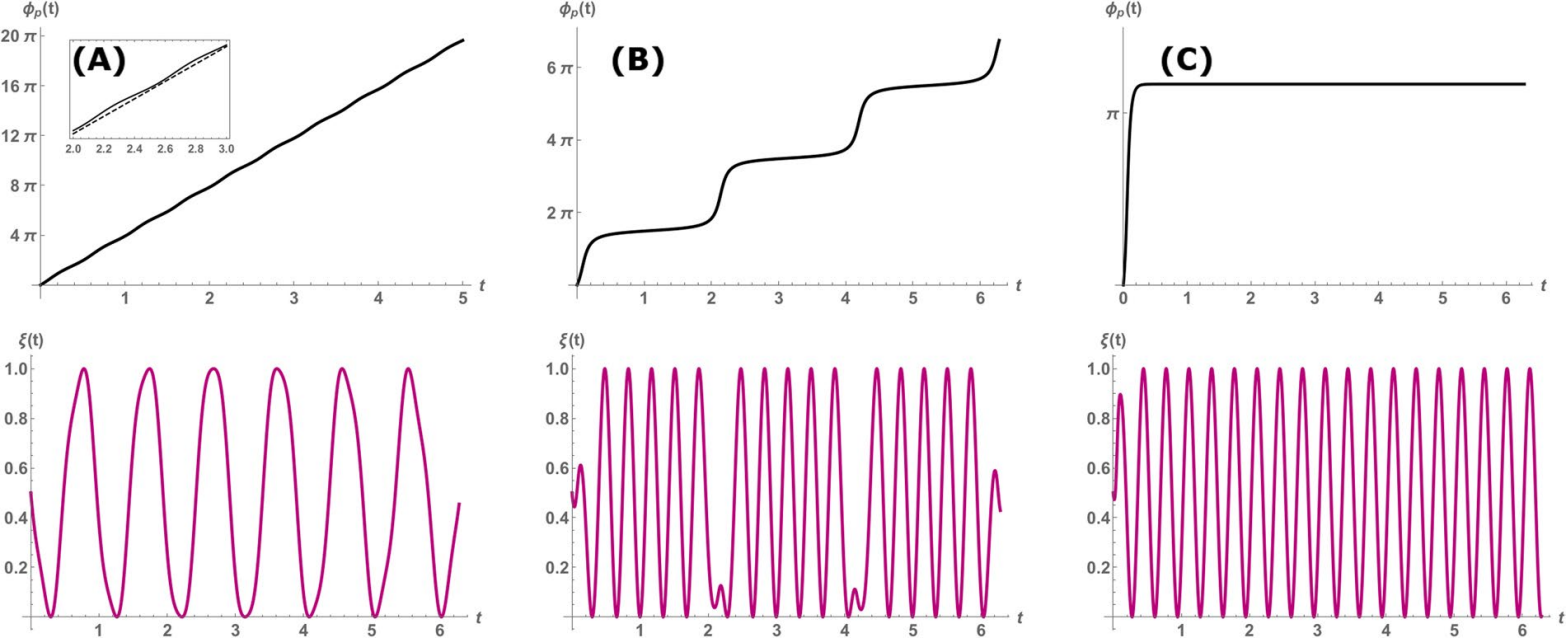
An example of the time evolution of the phase in Eq. 2 (top row) - and the resulting oscillation in *χ*(*t*) (bottom row), with *ω*_*c*_ = 2*π*, *ω*_*p*_ = 6*π*. (**A**) *Q* = 0.2, below threshold of entrainment at *Q* = 1, the cell beat with its spontaneous frequency *ω*_*c*_ as indicated by the quasi-linear increase in phase. Inset: comparison to linear slope (dashed) that shows large regions of slips and much smaller intervals of plateaus. (**B**) *Q* = 0.97 Intermittent periods of entrainment (plateaus) followed by fast “phase-slip” events. This corresponds to the “bursting” behavior observed in experiments^23^. (**C**) *Q* = 2, above the threshold of entrainment. The cell beats with the probe frequency *ω*_*p*_.

For large enough amplitude *A*_*p*_ the cell oscillates with the frequency of the probe. It is thus convenient to write $\chi (t)\sim A(t)\cos ({\omega }_{p}t+{\varphi }_{p}(t))$, where we introduce the time dependent amplitude (*A(t)*) and phase (*ϕ*_*p*_*(t)*) of the paced cell (see Sec. A in the SI). If both phase and amplitude change with a time scale much longer than that of a single beating cycle (as found experimentally), and when the probe frequency is not very different from the cell frequency (*ω*_*p*_ ~ *ω*_*c*_), we insert the equation for *χ*(*t*) into Eq. 1 and apply the method of averaging^41^ to derive the following dynamical equation for the phase:

2 $${\dot{\varphi }}_{p}={\rm{\Delta }}\omega -{f}_{s}\,\cos ({\varphi }_{p})$$

where we introduce the detuning ${\rm{\Delta }}\omega ={\omega }_{c}-{\omega }_{p}$, and define the scaled amplitude ratio ${f}_{s}={A}_{p}/(2{\omega }_{p}{A}_{s})$ (see Sec. B in the SI). Equation 2 is the famous Adler equation, which was first derived in the context of electrical oscillators^42^. Integrating both sides, one obtains an analytical expression for the phase:

3 $${\varphi }_{p}=2\,\arctan (\sqrt{\frac{Q-1}{Q+1}}\,\tanh [\frac{1}{2}\sqrt{{Q}^{2}-1}{t}^{\ast }+{\varphi }_{0}])$$

where we define the reduced time ${t}^{\ast }={\rm{\Delta }}\omega t$, and the dimensionless coupling strength $Q={f}_{s}/{\rm{\Delta }}\omega$ whose value determines whether the cell beats with its spontaneous frequency or with that of the probe. In Fig. 1 we plot the phase (top row), and the corresponding oscillations in *χ*(*t*) (bottom row) vs. time. For *Q* > 1, corresponding to strong pacing or a small difference in frequencies, the cell is synchronized to the probe and beats with frequency *ω*_*p*_. The phase given by Eq. 3 exponentially relaxes to a constant value, with a time scale $\tau  \sim {({\rm{\Delta }}\omega \sqrt{(|{Q}^{2}-1|)})}^{-1}$ (see Fig. 1C). However, for $|Q| < 1$ the phase dynamics is periodic in time, with a period of 2*π/τ*. Each period is step-like, and comprises fast “jumps” (slips) followed by a relatively shallow plateau. The slips correspond to an interval in which the cell beats spontaneously with *ω*_*c*_, while the plateaus correspond to the cell temporarily entrained by the probe, and beating with *ω*_*p*_.

It is important to note that in the entire range 0 ≤ *Q* ≤ 1, the apparent beating frequency is not merely the average frequency of the cell and probe, but reflects the dynamical process of switching between the two, which is solely determined by the coupling strength *Q*. We next analyze the duration of both the plateaus and slips of the beating phase in the regime of 0 ≤ *Q* ≤ 1). If $|Q|\ll 1$ (corresponding to either a weak probe or a large difference in frequencies), the time interval of the plateau goes to zero as ~*Q*^−1/2^, and the cell beats with its own natural frequency *ω*_*c*_ (see the linear slope in Fig. 1A). However, as $|Q|$ increases to values nearer to unity, *τ* increases and with it the duration of each period. The duration of each plateau scales as $\sim {\sqrt{1-Q}}^{-1}$ (see Sec. B in the SI), while the slips at the edges become steeper and exist for relatively short time intervals. This phase-slipping corresponds to the “bursting” behavior observed in experiments (see Fig. 1B). Comparison of these predictions with future measurements will allow quantitative verification of the simple model of the entire cell as a non-linear oscillator.

### Dynamical transition from spontaneous to entrained beating

Experiments show that the time required to mechanically pace cells is of the order of minutes, much longer than the beating frequency^23^. We can account for this by considering the biological adaptation of the cell in response to applied mechanical forces.

Our model considers the temporal delay between the time the force acts on the cell adhesions and their influence on the acto-myosin units (and related biochemistry) within the cell. This effect is included in a phenomenological, coarse-grained manner by modifying Eq. 1 to include a time-delayed response of the cell to the pacing force (first applied at *t* = 0):

4 $$\ddot{\chi }+\rho \dot{\chi }+\lambda {\dot{\chi }}^{3}+{\omega }_{c}^{2}\chi ={A}_{p}(1-{e}^{-t/{\tau }_{a}})\cos ({\omega }_{p}t)$$

where *τ*_*a*_ is the characteristic time for adaptation. For short times ($t\ll {\tau }_{a}$ the pacing does not affect the cell, while for long times ($t\gg {\tau }_{a}$) biological changes within the cell couples the oscillating degree of freedom *χ*(*t*) (be it acto-myosin displacement, and/or calcium concentration) responsible for beating to the displacement of the adhesions by the external force. If we assume that the time-scale for adaptation is much longer than a single beating cycle (i.e. ${\tau }_{a}\gg {\omega }_{p}^{-1}$) we can derive a modified Adler equation:

5 $${\varphi }_{p}({t}^{\ast })=1-Q(1-\frac{{\tau }_{a}}{{t}^{\ast }}(1-{e}^{-{t}^{\ast }/{\tau }_{a}}))\cos ({\varphi }_{p}(t\ast ))$$

As shown in Sec. C of the SI, when the external force is applied, the phase initially oscillates and then reaches a steady state value given by ${\dot{\varphi }}_{p}=0$ in Eq. 2. For Eq. 3 the transition from oscillatory to monotonic relaxation of the phase happens when the argument inside the hyperbolic tangent changes from pure imaginary to real. In Eq. 5, this happens when the term multiplying the cosine equals 1, that is:

6 $$\frac{(Q-\mathrm{1)}}{Q}=\frac{{\tau }_{a}}{{t}_{tr}}(1-{e}^{-{t}_{tr}/{\tau }_{a}})$$

If *Q* ~ 1, the left side of Eq. 6 goes to zero. For the right side, this implies that the transition time must be large (i.e. ${t}_{tr}\gg {\tau }_{a}$) to satisfy the equation. For this case, one can write the scaling argument:

7 $${t}_{tr} \sim \frac{{\tau }_{a}Q}{(Q-\mathrm{1)}}$$

The time required to pace the cell grows linearly with the adaptation time *τ*_*a*_ and diverges close to the transition point *Q* = 1. For a coupling strength close to the critical coupling (*Q* = 1) the time to pace the cell can becomes much longer than the characteristic adaptation time *τ*_*a*_ due to the non-linear dynamics. This scaling can be made more accurate for all values of *Q* by introducing a correction *w(Q*, *τ*_*a*_) (see Sec. C of the SI) which is zero for $Q\to 1$. With the correction, we find that the transition time can be reduced by increasing *Q*, which corresponds to increasing the probe amplitude *A*_*p*_ or by decreasing the detuning Δ*ω*. A comparison of our analytical prediction with numerical estimation of *t*_*tr*_ is presented in the Sec. C of the SI.

### Noise effects on beating and synchronization

Experiments show that although an isolated, beating cell has a well-defined average frequency; there is some measurable noise in both the amplitude and phase of the oscillations^43^. As a result, the beating amplitude fluctuates and the time interval between peaks is also variable.

In this section, we show how noise modifies the distribution of beating phases in steady-state, and how noise results in the decorrelation of the beating phase at long times.

To include the effects of noise, we introduce a stochastic force *χ*_*s*_(*t*) in Eq. 1:

8 $$\ddot{\chi }-\rho \dot{\chi }+\lambda {\dot{\chi }}^{3}+{\omega }_{c}^{2}\chi ={A}_{p}\,\cos ({\omega }_{p}t)+{\chi }_{s}(t)$$

Since we are interested in the long time response of the cell, we take *χ*_*s*_(*t*) to have the properties of white noise, with mean $\langle {\chi }_{s}(t)\rangle =0$ and temporal correlation $\langle {\chi }_{s}(t){\chi }_{s}(t^{\prime} )\rangle \sim 2{D}^{\ast }\delta (t-t^{\prime} )$) with D^*^ a measure for the magnitude of correlations. For a weak pacing force $Q\ll 1$, we expect that on average, the cell beats with the natural frequency *ω*_*c*_. We therefore use the reference point of oscillations at *ω*_*c*_ and write $\chi (t)\sim A(t)\cos ({\omega }_{c}t+{\varphi }_{c}(t))$, where *A(t)* and *ϕ*_*c*_*(t)* are now the stochastic amplitude and phase respectively. For small noise, the amplitude has small fluctuations around a steady-state average value^35^, and we can derive a Langevin equation for the phase (see Sec. D of the SI):

9 $$\begin{array}{l}{\dot{\varphi }}_{c}=\alpha \,\cos ({\varphi }_{c}-\beta )+{\bar{\chi }}_{\varphi }(t)\end{array}$$

where ${\bar{\chi }}_{\varphi }$ is the noise averaged over a beating cycle and *α* and *β* are functions of the two frequencies *ω*_*c*_ and *ω*_*p*_. For ${\omega }_{p}\sim {\omega }_{c}$, these are approximated by:

10 $$\begin{array}{cc}\alpha \approx {f}_{s}(1-\frac{1}{2}\frac{{\rm{\Delta }}\omega }{{\omega }_{c}}) & \beta \approx \pi \frac{{\rm{\Delta }}\omega }{{\omega }_{c}}\end{array}$$

To predict the dynamics of the phase, we derive from the Langevin equation for the stochastic phase a Fokker-Planck equation for the probability density *p*(*ϕ*_*c*_, *t*; *ϕ*_*c*_, 0) which predicts the distribution of the phase *ϕ*_*c*_ at time *t*, given a known distribution of the phase at time zero.

11 $$\dot{P}=-\frac{\partial }{\partial {\varphi }_{c}}(\alpha \,\cos ({\varphi }_{c}-\beta )P)+D\frac{{\partial }^{2}P}{\partial {\varphi }_{c}^{2}}$$

with the parameter $D={D}^{\ast }/{({\omega }_{c}{A}_{s})}^{2}$ (see Sec. D of the SI).

**Figure 2:**
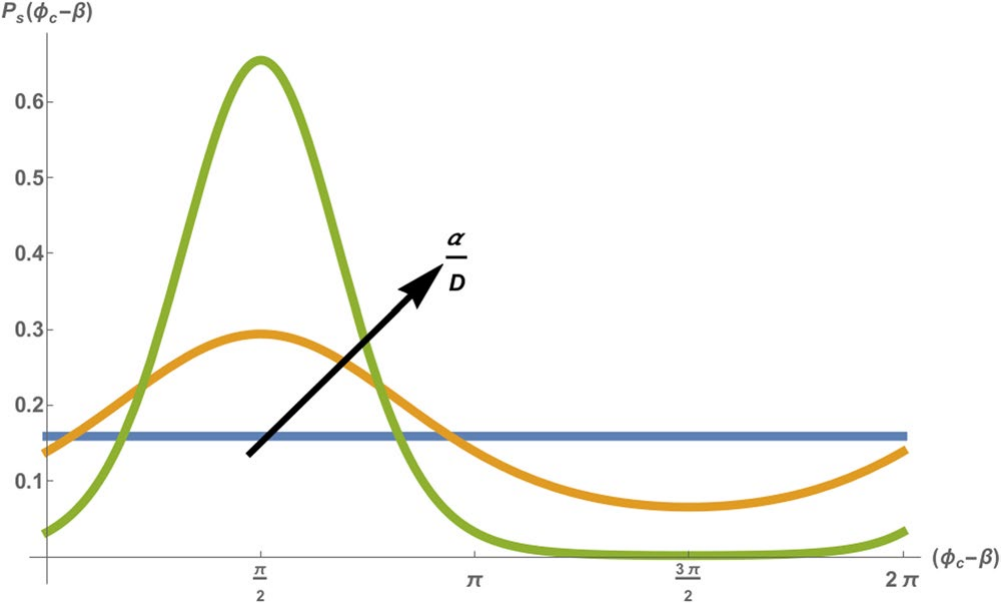
Stationary probability distribution, plotted as a function of the shifted phase (*ϕ* − *β*), for $D=0.5\,{s}^{-1}$, ${\rm{\Delta }}\omega =\pi \,{s}^{-1}$ and for pacing force *f*_*s*_ = 0 (blue), ${f}_{s}=0.5\,{s}^{-1}$ (orange) and ${f}_{s}=2\,{s}^{-1}$ (green).

Equation 11 describes a diffusion-like process of the phase *ϕ*_*c*_, with a drift^44^ due to a periodic potential $V({\varphi }_{c})=-\alpha \,\sin ({\varphi }_{c}-\beta )$. For long times ($t\gg 1/D$) we expect the probability density to relax to a steady-state distribution ${P}_{s}({\varphi }_{c})$ which is independent of the initial probability distribution. The steady state distribution is 2*π*-periodic and normalized to a single cycle:

12 $${P}_{s}({\varphi }_{c})=\frac{\exp (\frac{\alpha }{D}\,\sin ({\varphi }_{c}-\beta ))}{2\pi \,{I}_{0}(\alpha /D)}$$

where *I*_0_ is the modified Bessel function of the first kind. Eq. 12 is plotted in Fig. 2 for various values of probe amplitude (*f*_*s*_). In the absence of an external probe (*f*_*s*_ = 0) the potential is zero which yields a uniform distribution of the shifted phase (see Fig. 2) with a variance of $\langle {\varphi }_{c}^{2}\rangle ={\pi }^{2}\mathrm{/3}$. Therefore, each value of the phase has an equal probability, which predicts noisy oscillations. However, as the amplitude of the probe (*f*_*s*_) increases, the potential displays distinct extrema, which result in a narrowing of the distribution around an average phase $\langle {\varphi }_{c}\rangle =\beta +\pi \mathrm{/2}$. The width of this distribution (and hence the mean square fluctuations of the steady-state phase) scales as *D/α*. This means that the probe serves as an external force that keeps the phase trapped in a local minimum, effectively reducing the apparent noise in beating. This result is even more pronounced in the case of a strongly paced cell, which we address in Sec. D of the SI.

## Discussion

In this paper, we have predicted various dynamical transitions that can occur when a probe or another cell is mechanically coupled to a beating cardiomyocyte using a non-linear, one dimensional model for active oscillations. It is important to note that the results are generic for any spontaneously oscillating degree of freedom, be it the changes in cell length or the apparent calcium concentration. There are several options for the coupling of the external mechanical force to the contraction of the cell, depending on the scenarios listed in the Introduction section and detailed in the SI:

1. The mechanical force directly pace the acto-myosin contractile unit (as suggested by Jülicher and Prost^29^), by pulling on the sarcomeres via the adhesion points connecting them to the substrate. This then varies the binding and unbinding dynamics of acto-myosin complexes, as well as the dynamics of force application.
2. The mechanical force couples to the sarcolemma (or SR) via the cellular adhesions, and the stress applied is translated to a tension in the sarcolemma (or SR). This tension can result in modulation of stretch sensitive proteins which, in turn, change calcium concentration in the cytosol and modulate contractility.
3. A third scenario is that the mechanical force is coupled to acto-myosin, which is sequentially coupled to calcium concentration. The mechanical pacing releases calcium normally bound to actin (via troponin) back into the cytosol, effectively changing the calcium concentration. This scenario involves cell contractility as a necessary mediator in entraining calcium oscillations.

The results by Nitsan *et al*.^23^ suggest that inhibiting contractility impairs the ability of cells to synchronize to mechanical forcing, therefore favoring scenario (3). The molecular details of the electro-mechanical coupling, although very interesting, are beyond the scope of this paper.

Another important point is that our model applies to experiments performed on neonatal cardiomyocytes, which exhibit spontaneous beating. In contrast, adult cardiomyocytes do not tend^45^ to beat spontaneously. In the context of our model, the difference between neonatal and adult cells is reflected in the change of the sign of the feedback term (*ρ*), from negative to positive, for which there are no spontaneous oscillations (see Sec. A1 of the SI). For the first scenario suggested (mechanical pacing of the contractile machinery), this could be caused by an increase in dissipation of energy due to the cytoskeleton becoming more dense and ordered in adult cells. For the second scenario (mechanical pacing of calcium release), the inversion of sign of the feedback *ρ* can be attributed to changes in expression of ionic pumps and channels embedded in the SR. Neonatal cardiomyocytes are much more plastic than adult cardiomyocytes, which allows larger scale variations in their structure and biochemical pathways^37,46^. However, as cells age, the cytoskeleton matures and the protein concentration varies, making both processes plausible explanations for the loss of spontaneous beating in adult myocytes.

The non-linear nature of the model is essential to account for the experimental results since a simple linear oscillator model coupled to an external force would result only in superposition of the cell and probe frequencies; neither bursting nor entrainment would be observed^38^. Our quantitative predictions for the dependence of the bursting regime on the probe amplitude and frequency (as expressed in our coupling parameter *Q*) suggest future experiments. Comparison of our predictions in Sec. 2 and such experiments are a stringent test of the applicability of the simple non-linear oscillator model to the beating of an entire cardiomyocyte. We further suggest that future experiments will measure the dependence of the “bursting” regime on the amplitude of the probe. The results, when compared to our predictions in Sec. 2 for the scaling of the phase slips with the coupling parameter *Q*, can offer quantitative validation of the Adler description for paced cardiomyocytes.

We suggest that the time required for entrainment (minutes) may originate in an adaptive change in the ability of the cell to sense and react to the external force. This adaptation can be the result of subtle structural changes within the cytoskeleton, change in expression of ion-channels and ion-pumps on the SR^47–50^ or release of growth factors that reinforce sensing or recruit protein complexes associated with sensing^51^. These biological processes usually require several minutes to be established, and any combination of those may result in the coarse-grain time-scale *τ*_*a*_ in our model. Molecular details require many more experiments and are outside the scope of this work. Our coarse grained model predicts quite generally, that the time required to entrain a cell should decrease with an increase in probe amplitude (see Sec. 3). Although long time scales have been measured, those have not been quantified as a function of the probe amplitude and frequency. Comparing such future experiments with the theory presented here will shed light on the adaptation assumption and its more detailed properties. Our theory does not account for the even longer timescale for returning to the spontaneous frequency once the pacing has stopped. This would imply another level of adaptivity, which might involve changing *ω*_*c*_ itself.

In experiments, the beating of isolated cardiac cells has a stochastic component in both amplitude and phase. The stochasticity in phase is manifested in the isolated events of skipping or adding a beat, and has important biological implications. Such noise might stem from the inherent length or elasticity differences among different sarcomeres^52^, or from the spatial disorganization of said sarcomeres within the cell^18,46^.

For a paced cell, the noise only produces small fluctuations in phase since the amplitude of the pacing force is much larger, creating a minimum in the energy landscape for the phase. This is analogous to the restricted diffusion (due to noise) of a particle in the vicinity of an optical trap^53^. We suggest that experiments quantify the range of the beating phase before and after entrainment. According to our theory, this should allow an estimate of the effective noise relevant to the beating dynamics. If the amplitude of the noise decreases even after pacing is stopped, this might indicate that the intrinsic noise level is changed by entrainment. This may serve as another indication for the existence of an adaptation mechanism within the cell.

Our theory provides an intuitive explanation of the dynamics of mechanical synchronization. At the tissue level, this is relevant to the ability of cells to coordinate their beating, which is important for the development of an embryonic heart^54^ and for the susceptibility of heart cells to various pathological conditions. The quantitative experiments we suggest can serve to verify the validity of our coarse grained model to the phenomenon of mechanical entrainment. Evaluating the ability of cells to react and respond to mechanical pacing might also prove useful in early detection of beating irregularities and provide guidelines for the design of future treatment.

## Electronic supplementary material

Supplementary information
