# Supplementary material for: Theory of frequency response of mechanically driven cardiomyocytes
Source: Sci Rep. 2018 Feb 2;8:2237. doi: 10.1038/s41598-018-20307-2 (PMC5797104; doi:10.1038/s41598-018-20307-2)
Supplement: Supplementary file 1 — Supplementary information [file 41598_2018_20307_MOESM1_ESM.pdf]

Theory of frequency response of mechanically driven cardiomyocytes - Supplementary Information

Ohad Cohen*, Samuel A. Safran

Dept. Chemical and Biological Physics, Weizmann Institute of Science, Rehovot, IL, 76100

December 4, 2017

A Biophysical origin and dynamics of spontaneous beating

We start with the generic equation for the non-linear Rayleigh oscillator [1]:

$$\gamma\ddot{\chi} + r\dot{\chi} + \Lambda\dot{\chi}^3 + k\chi = f_{ext}(t) \tag{A.1}$$

We discuss here three possible scenarios that motivate the biological origin of this equation for the oscillating degree of freedom $\chi(t)$:

1. $\chi(t)$ represents the physical displacement of the contractile machinery (sarcomeres) coupled to the deformations induced by the probe ($f_{ext}(t)$). This model was discussed in detail by Jülicher and Prost [2, 3] in the context of acto-myosin displacement within the sarcomere. The elastic response of the acto-myosin bundle is manifested in the spring constant k. The first order friction coefficient, ρ accounts for both dissipation of energy via the viscous environment as well as for the input of energy by the ATP dependent activity of myosin. Therefore, unlike purely damped oscillators, ρ can have a negative sign (when activity dominates friction) which allows for spontaneous contraction oscillations with frequency $\omega_c = \sqrt{k/\gamma}$. If only linear terms are kept, energy input (the case of $r < 0$) would result in a diverging amplitude. To maintain stability, the overall energy input must dissipated by a higher order friction term $\lambda > 0$, and is related by Jülicher and Prost to the loading rate of myosin heads. Lastly, $f_{ext}(t)$ is the mechanical force applied to the contractile unit via the cellular adhesions that connect the sarcomeres to the substrate, such as focal adhesions at the cell edges [4] or integrin-mediated adhesions (by costameres) along the line of sarcomeres [5].

2. $\chi(t)$ represents the ionic calcium concentration in the cytosol, and the external force applies tension to the either the sarcolemma, or directly to the sarcoplasmic reticulum (SR) - affecting calcium release. In this context, ρ relates to auto-catalytic activation of ryanodine receptors (RyR) on the SR, and the higher order restoring term $\lambda > 0$ represents deactivation of RyR (which is still not fully understood) once a certain threshold of cytoplasmic concentration is reached [6, 7]. The spring constant k entails numerous mechanisms such as SR Ca^2+-ATPase activity, sarcolemma Na^+-Ca^2+ pumps and mitochondrial Ca^2+ uniports, all of which working to reduce calcium concentration to baseline levels. Lastly, the external force f_{ext} is coupled to the sarcolemma (or the SR), through integrin adhesions [5]. The tension applied to the sarcolemma (or the SR membrane) modulates the activity of mechano-sensitive protein complexes that modulate calcium release from SR [8].

3. $\chi(t)$ represents the calcium concentration as in the second scenario, but the external force is coupled via the adhesions of the substrate to sarcomeres. In this case, the deformations in the substrate by the probe

modulate the binding kinetics of actin-myosin, which, in turn, either apply further tension to the stress sensitive sarcolemma or SR, or reduce the amount of calcium bound to troponin at rest. This modulates the effective ionic concentration, and further paces the calcium concentration $\chi(t)$. In this scenario, contractility is a crucial intermediate step in entrainment of calcium.

For each of these cases, the acceleration term Eq. A.1, proportional to γ, is not at all related to inertia (mass) because both the cell interior and exterior are aqueous solutions where frictional dissipation is dominant and inertia is negligible [9, 10]. Rather, this term originates in the dynamical active process of binding and unbinding of myosin motors to actin filaments (first scenario), or from the dynamics of opening and closing of RyR channels (second and third scenarios). The separation of the time scales associated with binding/unbinding of myosin, or opening/closing event of RyR, and the time scale of apparent oscillations yields an "effective mass" $\gamma > 0$ term that is a function of the different kinetic rate constants in the system (as shown in detail in Ref. [3]).

To demonstrate this, we study a simple system that couples contraction of a sarcomere ($x(t)$) to the actin bound myosin concentration ($\mu(t)$) (this is analogous to the derivation of Jülicher and Prost [2, 3], but perhaps more transparent):

$$\dot{\mu} = \kappa^+ \mu_{tot} - (\kappa^+ + \kappa^-)\mu + a_1 \dot{x} \tag{A.2}$$

$$\eta_f \dot{x} + \Lambda \dot{x}^3 = a_2 \mu - kx \tag{A.3}$$

Here, κ^+/κ^- are binding unbinding rates of myosin to actin, μ_{tot} is the overall myosin concentration available for binding in a sarcomere, η_f is the friction coefficient and k is the elastic response of the sarcomere modeled as a damped spring. A non-linear friction term proportional to $\Lambda > 0$ is introduced, for reasons explained below. In this simple picture, the contraction increases the overlap region between myosin and actin, allowing for more myosin heads to bind feeding back into the myosin rate equation (as expressed by the term proportional to a_1). The force generated by the sarcomere is proportional to the number of bound myosin heads (hence a_2). The solution for Eq. A.2 for the myosin concentration, as a function of the general, time dependent displacement, is given in terms of the Green's function $G(t)$:

$$\mu(t) = \mu_h(t) + a_1 \int_{-\infty}^{t} G(t - t')\dot{x}(t')dt' \tag{A.4}$$

where $\mu_h(t) = \mu_0 \exp(-(\kappa^+ + \kappa^-)t)$ is the solution of the homogeneous equation and μ_0 some arbitrary initial condition.

The myosin binding and unbinding rates are typically much faster ($\sim 0.01 - 0.1$ Hz) [11] than the time-scale of associated with oscillations ($\omega_c \sim 1$ Hz). In the context of the above model, this implies that there are numerous binding/unbinding events of myosin heads in one contraction cycle of the sarcomeric

unit. The Green's function for Eq. A.4 is given by $G(t-t') = \exp(-(\kappa^+ + \kappa^-)(t-t'))$ which is sharply peaked around $t' = t$ (since $k^+, k^- << \omega_c$, the time scale for changes in displacement). If the Green's function is sharply peaked about the time $t' = t$, we can expand the slowly varying velocity $\dot{x}(t')$ about $t' < t$, and introduce it back into Eq. A.4:

$$\mu(t) = \mu_h(t) + a_1\dot{x}(t)\int_{-\infty}^{t} G(t-t')dt' + a_1\ddot{x}(t)\int_{-\infty}^{t}(t'-t)G(t-t')dt' \quad \text{(A.5)}$$

The feedback between the velocity $\dot{x}(t)$ and the position $x(t)$ is proportional to the integral of the Green's function (zeroth order moment). The inertial term is associated with the first moment of the Green's function, which accounts for force accumulation due to the positive feedback between contraction and the increase of bound myosin. In our simple picture, the Green's function is $G(t-t')$ =exp$(-(\kappa^+ + \kappa^-)(t-t')$, which is indeed sharply peaked around $t' = t$. Using this result, the equation for myosin concentration in our example scales as:

$$\mu(t) \sim \frac{\kappa^+\mu_{tot}}{(\kappa^+ + \kappa^-)} + \frac{a_1}{(\kappa^+ + \kappa^-)}\dot{x(t)} - \frac{a_1}{(\kappa^+ + \kappa^-)^2}\ddot{x(t)} \quad \text{(A.6)}$$

which, when introduced back into Eq. A.3 for the displacement yields:

$$\gamma^*\ddot{x} + r^*\dot{x} + \Lambda\dot{x}^3 + kx = 0 \quad \text{(A.7)}$$

With $\gamma^* = a_1/(\kappa^+ + \kappa^-)^2$ and $r^* = (\eta_f - a_1/(\kappa^+ + \kappa^-))$. Note that the activity of myosin results in an inertial term with an effective mass γ^*. The "mass" γ^* is related to the cooperative dynamics of myosin binding and the active force they apply to contract the sarcomere. Note that r^* can be negative if the input of energy to the oscillator from the activity dominates over friction (i.e. - $\eta_f < a_1/(\kappa^+ + \kappa^-)$). In that case, the sarcomere will spontaneously contract. Note that the non-linear frictional dissipation term ($\Lambda > 0$) must be present since otherwise the input of energy for the case where $r* < 0$ will causes diverging oscillation amplitudes.

With the inertial term derived, and a higher order dissipation term $\Lambda > 0$ introduced to suppress unbounded growth, the system displays spontaneous oscillations for values $r < 0$. This is inherently different from a regular damped oscillator ($r > 0$), where any dissipation causes any initial displacement or velocity to eventually decay to 0. In the absence of external pacing $f_{ext}(t) = 0$, for the case where the spring constant is large (stiff spring) relative to the dissipative terms (r, $\Lambda \ll k$), one solves Eq. A.1 and finds that the system oscillates with the natural frequency $\omega_c = \sqrt{k/\gamma}$. Therefore, we insert the solution $\chi(t) = A(t)\cos(\omega_c t)$ into Eq. A.1 to find:

$$\begin{aligned}\gamma((\ddot{A} - A\omega_c^2)\cos(\omega_c t) - 2\dot{A}\omega_c\sin(\omega_c t) + r(\dot{A}\cos(\omega_c t) - A\omega_c\sin(\omega_c t))\\ +\Lambda(\dot{A}\cos(\omega_c t) - A\omega_c\sin(\omega_c t))^3 + kA\cos(\omega_c t) = 0\end{aligned} \quad \text{(A.8)}$$

For long times, we expect the amplitude to relax to its steady-state value, A_s. The steady-state is defined by the oscillator state when the amplitude does

not change in time. We therefore set $\dot{A}$ and $\ddot{A}$ equal to zero, and collect terms of sine and cosine:

$$A_s(k - \gamma\omega^2)\cos(\omega_c t) + A_s\omega_c(r - \Lambda A_s^2\omega_c^2\sin^2(\omega_c t))\sin(\omega_c t) = 0 \tag{A.9}$$

For Eq.A.9 to be valid for all times, the terms multiplying the sine and cosine separately must equate to zero:

$$\begin{aligned} \frac{A_s}{2}(k - \gamma\omega^2) = 0 \\ \frac{A_s\omega_c}{2}(r - \frac{3}{4}\Lambda A_s^2\omega_c^2) = 0 \end{aligned} \tag{A.10}$$

The first condition determines the oscillation frequency in steady-state, and the second determines the average steady state amplitude $A_s = \sqrt{4r/3\Lambda\omega_c^2}$ as shown in the paper.

B Dynamics of a paced cell

B.1 Derivation of the Adler equation

We now introduce periodic forcing by an external probe/cell $f_{ext}(t) = F\cos(\omega_p t)$, which oscillates at a frequency that is, in general, different from the spontaneous beating frequency ω_c. Rescaling all the coefficients of Eq. A.1 by the effective mass γ, so that $\rho = r/\gamma$, $\lambda = \Lambda/\gamma$, $A_p = F/\gamma$ (same notation as in the main text) we get:

$$\ddot{\chi} + \rho\dot{\chi} + \lambda\dot{\chi}^3 + \omega_c^2\chi = A_p\cos(\omega_p t) \tag{B.11}$$

For the entrained regime, where the system oscillates at the probe frequency ω_p, we write the solution in complex form:

$$\chi(t) = a(t)e^{i\omega_p t} + a^*(t)e^{-i\omega_p t}, \quad a(t) = A(t)e^{i\phi_p(t)}, \quad a^*(t) = A(t)e^{-i\phi_p(t)} \tag{B.12}$$

where $A(t)$, $\phi_p(t)$ are respectively, the amplitude and phase of the oscillating displacement $\chi(t)$.

Since the observed time that it takes a spontaneously beating cell to synchronize with the probe is much longer than the characteristic beating time (15 minutes compared to a time scale of 1 second, corresponding to $\sim$ 1 Hz oscillations) we use the method of averaging – where we average over the slowest mode of oscillation [12]. For an arbitrary function of time $g(t)$ with period T, this is written as:

$$\langle g(t)\rangle = \frac{1}{T}\int_{-\frac{T}{2}}^{\frac{T}{2}} g(t)e^{it/T}\, dt \tag{B.13}$$

Inserting Eq. B.12 into Eq. B.11, and averaging over the slowest mode (with a period $2\pi/\omega_p$) we write the dynamics of $a(t)$ as:

$$
\begin{aligned}
&(\ddot{a} + 2i\omega_p\dot{a}) - (\omega_p^2 - \omega_c^2)a - \rho(\dot{a} + i\omega_p a) + \tfrac{\lambda}{4}\left(3\dot{a}^2(\dot{a^*} - i\omega_p a^*)\right. \\
&\quad \left. +6\dot{a}(aa^*\omega_p^2 + i\omega_p a\dot{a^*}) - 3a^2(\dot{a^*}\omega_p^2 - i\omega_p^3 a^*)\right) - A_p = 0
\end{aligned}
\tag{B.14}
$$

We now define the time scale $a/\dot{a} \sim \tau$ which is much longer than the time scale for oscillations $1/\omega_p$ (since the amplitude and phase are slowly varying). Because this time scale is long, we consider only terms up to order $1/\tau$ in Eq.B.14. All terms higher order than $\dot{a}/a$ are neglected in the long-time limit. Using the definitions in Eq. B.12 and comparing real and imaginary terms, we obtain two dynamical equations for the phase amplitude $A(t)$ and phase $\phi_p(t)$:

$$
\begin{aligned}
2\omega_p\dot{A} &= \rho\omega_p A - \frac{3}{4}\lambda\omega_p^3 A^3 - A_p\sin(\phi_p) \\
2\omega_p A\dot{\phi_p} &= (\omega_c^2 - \omega_p^2)A - A_p\cos(\phi_p)
\end{aligned}
\tag{B.15}
$$

For weak forcing A_p, the dynamical equation for the amplitude becomes independent of phase, and the steady-state amplitude has a similar form to that of a spontaneously beating cell, i.e. -$A_s = \sqrt{(4\rho/3\lambda\omega_p^2)}$ (where ω_c is replaced by ω_p due to the change in frame of reference).

We scale the probe amplitude (A_p) by the amplitude of spontaneous oscillations (A_s) and define the scaled probe amplitude $f_s = A_p/(2\omega_p A_s)$ as in the main text. Focusing on the case where the spontaneous frequency and the probe frequency are not very different (i.e. - $\omega_c \sim \omega_p$) we obtain the Adler equation (Eq. 2) as presented in the text. Note that in the experiments performed by Nitsan et al. [13] the amplitude ratio (equivalent to f_s) was kept fixed, so that the beating amplitude of the cell and the beating amplitude generated by the probe in the vicinity of the cell were roughly equal ($A_s \sim A_p$). According to our theory, this implies that the transition from entrained beating to bursting should occur when the frequency of the probe is roughly twice the frequency of the cell, the same order of magnitude as observed by Nitsan et al.[13].

B.2 Solving the Adler equation

First, we rescale time by the detuning $\Delta\omega = (\omega_c - \omega_p)$, so that $t^* \equiv \Delta\omega t$. This allows us to write the dimensionless form of Eq. 2:

$$
\dot{\phi_p(t^*)} = 1 - Q\cos(\phi_p(t^*)) \tag{B.16}
$$

where we define the coupling strength $Q = f_s/\Delta\omega$ as in the main text. This dynamical equation has two bifurcation points, $Q^{cr} = \pm 1$ where the steady-state solution change their character. For $|Q| \geq 1$ the equation has a steady-state solution corresponding to $\phi^{ss} = \arccos(Q^{-1})$. This describes the regime where cell beating is entrained to the probe frequency ω_p. However, for $|Q| \leq 1$ there is no steady state solution, and the phase grows with time. This corresponds to a beating cell not fully entrained to the probe. In the limit of very weak forcing, or a large frequency difference $\Delta\omega$, the phase grows linearly in time, which

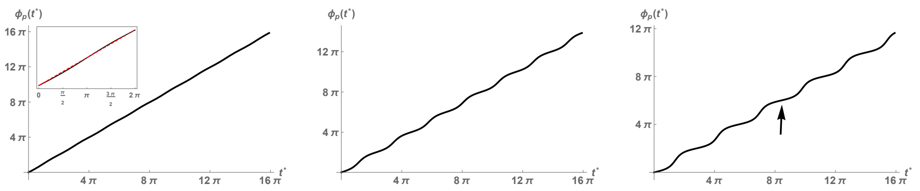

Figure 1: phase ϕ_p as a function of reduced time t^*, in the "bursting" regime ($|Q| < 1$) for: (A) Q=0.1 - Inset: comparison with a linear slope (dashed, red), (B) Q=0.5 and (C) Q=0.7, arrow pointing at "mid-point" of the quasi-entrainment regimes.

means that the cell beats with its own spontaneous frequency ω_c. However, for a value of Q close to the bifurcation point ($|Q| \to 1$) we expect the cell to be entrained most of the time, with occasional periods of non-entrained behavior (which, for now, we assume nothing about).

To find an expression for the phase ϕ_p, we integrate Eq. B.16 for the two regimes of Q. This yields:

$$\begin{aligned} \phi_p = & \quad 2\arctan\left(\sqrt{\tfrac{Q-1}{Q+1}}\tanh\left[\pi\tfrac{t^*}{\tau}+\phi_0\right]\right), \qquad |Q|>1 \\ \phi_p = & \ -2\arctan\left(\sqrt{\tfrac{1-Q}{Q+1}}\tan\left[\pi\tfrac{t^*}{\tau}+\phi_0\right]\right)+2\pi m, \quad |Q|<1 \end{aligned} \tag{B.17}$$

where we define the characteristic time $\tau = 2\pi/(\Delta\omega\sqrt{(1-Q^2)})$.For $|Q| < 1$, the square roots are imaginary which transforms the hyperbolic tangent to a regular one. Therefore, the equation becomes periodic in time with $m = 1, 2, 3...$ the period index, defined for the time intervals $\pi m - \tau/2 < t < \pi m + \tau/2$. Note that the time scale τ **cannot** explain the observed time of about 15 minutes required to entrain spontaneously beating cells, as this would imply Q extremely close to the critical value $Q^{cr} = \pm 1$, which is not necessarily the case in the experiments. In the main text, we discuss a more robust scenario for the long time needed to transition from spontaneous to entrained beating.

We now derive the time regime for $Q < 1$ for which the cell is approximately entrained and the time regime for which the cell beats spontaneously (with ω_c). To examine the periodic behavior of the solution in Eq. B.17 we arbitrarily set $\phi_0 = 0$ without loss of generality, and evaluate the time derivative of Eq. B.17.

$$\dot{\phi}_p = \frac{1-Q^2}{1+Q\cosh\left(\sqrt{1-Q^2}\ t^*\right)} \tag{B.18}$$

For $Q \to 0$ Eq. B.18 goes to unity, which is the limit where the cells beat with their spontaneous frequency. As Q is gradually increased, the phase shows distinct time regimes of intermittent slow and rapid changes (see Fig. 1). We show in the main text that the time regimes where the derivative $\dot{\phi}_p(t)$ is nearly zero corresponds to an entrained cell (beating with ω_p) while the time regimes where $\dot{\phi}_p(t) \geq 1$ corresponds to a non-entrained cell (beating with ω_c). To

estimate the fraction of each period for which the cell is approximately entrained (the temporal regime where $\dot{\phi}_p \approx 0$), we expand Eq. B.18 in time t^*, around any of the mid-points (see arrow in Fig. 1C) of the "slow" change in phase:

$$\dot{\phi}_p \approx (1-Q) + \frac{1}{2}Q(1-Q)^2\left(t^* - \frac{2\pi m}{\sqrt{1-Q^2}}\right)^2 \tag{B.19}$$

Thus in the vicinity of the mid-point, the change in phase is parabolic, and an estimate of the point of crossover (time t_c^*) from $\dot{\phi} \approx 0$ to $\dot{\phi} \approx 1$ occurs when the second term in Eq. B.19 approximately equals the first. This yields $t_c^* = \sqrt{2/\left(Q(1-Q)\right)}$, which for $Q \to 1$ goes to infinity; namely, the cell becomes entrained for the entire time of its beating cycle. In the opposite limit (small Q), we expand around the region where $\dot{\phi}_p \geq 1$ of any one cycle to find:

$$\dot{\phi}_p \approx (1+Q) + \frac{1}{2}Q(1+Q)^2\left(t^* - \frac{\pi + 2\pi m}{\sqrt{1-Q^2}}\right)^2 \tag{B.20}$$

Using an analysis similar to the one for $Q \to 1$, we see that when $Q \to 0$ almost all of the beating cycle is characterized by $\dot{\phi} \approx 1$ (beating with spontaneous frequency). By comparing the first and second term as before, we get $t_c^* = \sqrt{2/(Q(1+Q))}$ which diverges for $Q \to 0$. This means that in the limit of very weak pacing force, the fraction of the beating cycle for which $\dot{\phi}_p \approx 1$ is almost unity and the duration for which the cell is entrained is $(\dot{\phi}_p \approx 0)$.

C Dynamics of adaptive response

The experiments show that the time required for a cell to transition from spontaneous to entrained beating is about 15 minutes [13], which is two to three orders of magnitude longer than the duration of a single beating cycle (∼1 sec).

The observed long time for the transition from spontaneous beating may be due to biological adaptation needed to transmit mechanical information (probe-induced strain of the cellular adhesions) to the cytoskeleton, resulting in a change in the beating frequency. We therefore apply a phenomenological description since the biological details are not known and are beyond the scope of the theory presented in this paper. Below, we consider the case where the adaptation process is manifested in the ability of the cell to sense the periodic deformations by the nearby probe.

We start by modifying the pacing force in Eq. B.11 to include exponential dependence in time, with a characteristic time scale τ_a (related to the biological modifications within the cell):

$$\ddot{\chi} - \rho\dot{\chi} + \lambda\dot{\chi}^3 + \omega_c^2\chi = A_p(1 - e^{-t/\tau_a})\cos(\omega_p t) \qquad \text{(C.21)}$$

where τ_a is the adaptation time which experiments suggest, is much longer than that of a single beating cycle (i.e.$\sim 1/\omega_c,\ 1/\omega_p$). Anticipating that the steady-state will be entrained beating, we use a reference state where the phase $\phi_p(t)$ is relative to $\omega_p t$ and write $\chi(t) = A(t)\cos(\omega_p t + \phi_p(t))$, with $A(t)$ and $\phi_p(t)$ the time dependent amplitude and phase respectively. Following the same derivation as before (phase slowly varying, stiff spring compared to dissipative response) we average over a single cycle and separate real and imaginary parts as in Eq. B.14. This yields two dynamical equations for the phase and amplitude:

$$\begin{aligned} \dot{\phi}_p &= \frac{(\omega_c^2 - \omega_p^2)}{2\omega_p} - \frac{A_p}{2\omega_p A} M(t)\cos(\phi_p - \psi(t)) \\ \dot{A} &= \frac{1}{2}\left(\rho - \frac{3}{4}\lambda(\omega_p A)^2\right) - \frac{A_p}{2\omega_p} M(t)\sin(\phi_p - \psi(t)) \end{aligned} \qquad \text{(C.22)}$$

where the (time dependent) correction, due to the finite adaptation time τ_a are:

$$M(t) = \sqrt{\left[1 - \frac{\tau_a}{t}\left(1 - e^{-t/\tau_a}\right)\left(\frac{2 + (2\tau_a\omega_p)^2}{1 + (2\tau_a\omega_p)^2}\right)\right]^2 + \left[\frac{\tau_a}{t}\left(1 - e^{-t/\tau_a}\right)\left(\frac{2\tau_a\omega_p}{1 + (2\tau_a\omega_p)^2}\right)\right]^2}$$

$$\psi(t) = \arctan\left(\frac{\frac{\tau_a}{t}\left(1 - e^{-t/\tau_a}\right)\left(\frac{2\omega_p\tau_a}{1+(2\omega_p\tau_a)^2}\right)}{1 - \frac{\tau_a}{t}\left(1 - e^{-t/\tau_a}\right)\left(\frac{2+(2\omega_p\tau_a)^2}{1+(2\omega_p\tau_a)^2}\right)}\right) \qquad \text{(C.23)}$$

Note that if we take $\omega_p\tau_a \gg 1$ (since experiments suggest that the adaptation

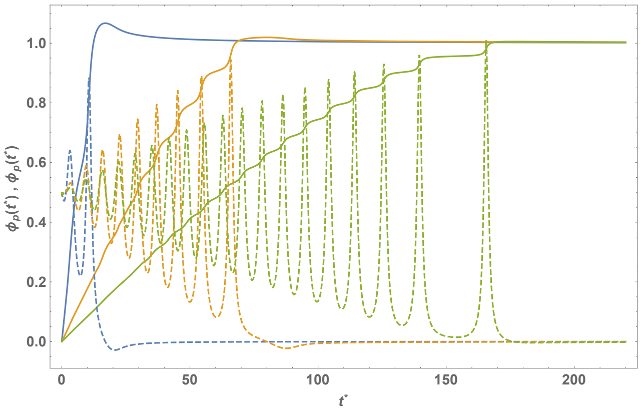

Figure 2: An example of the time dependent, normalized phase ϕ_p (solid) and its time derivative $\dot{\phi}_p$ (dashed), as a function of reduced time $t^* = \Delta\omega t$. Here $Q = 2$, and colors correspond to different values of the scaled adaptation time $\tau_a^* = \Delta\omega\tau_a$ with $\tau_a^* = 10$ (blue), $\tau_a^* = 50$ (orange) and $\tau_a^* = 100$ (green).

time is of the order of minutes, and the frequency is of the order of seconds), we can expand the corrections in the small parameter $(\omega_p\tau_a)^{-1}$and simplify:

$$\begin{aligned} M(t) &\approx 1 - \frac{\tau_a}{t}\left(1 - e^{-t/\tau_a}\right) \\ \psi(t) &\approx \arctan\left(\left(\frac{1}{2\omega_p\tau_a}\right)\frac{\frac{\tau_a}{t}\left(1 - e^{-t/\tau_a}\right)}{1 - \frac{\tau_a}{t}\left(1 - e^{-t/\tau_a}\right)}\right) \end{aligned} \tag{C.24}$$

For small values of probe amplitude A_p, the steady-state amplitude of the paced cell does not vary much from that of the spontaneously beating cell. If the amplitude reaches its steady-state value faster than the phase, the two equations of Eq. C.22 are decoupled, and we are left with a single dynamical equation for the phase:

$$\dot{\phi}_p = \frac{(\omega_c^2 - \omega_p^2)}{2\omega_p} - f_s\left(1 - \frac{\tau_a}{t}\left(1 - e^{-t/\tau_a}\right)\right)\cos(\phi_p - \psi(t)) \tag{C.25}$$

From Eq. C.24 we see that when the adaptation time is much longer than the beating time $\omega_p\tau_a \gg 1$, the function $\psi(t)$ drops to zero (from its initial value of $\pi/2$ at $t = 0$ in a time of order ω_p which is much smaller than the adaptation time. We thus consider times greater than this value and set $\phi_p(t)$ to be zero in Eq. C.25. For simplicity of notation, we consider the case of small frequency differences and write:

$$\dot{\phi}_p = \Delta\omega - f_s\left(1 - \frac{\tau_a}{t}\left(1 - e^{-t/\tau_a}\right)\right)\cos(\phi_p) \tag{C.26}$$

which is the modified Adler equation presented in the paper. One can immediately see that for short times $t \ll \tau_a$ the pacing is effectively zero, and for

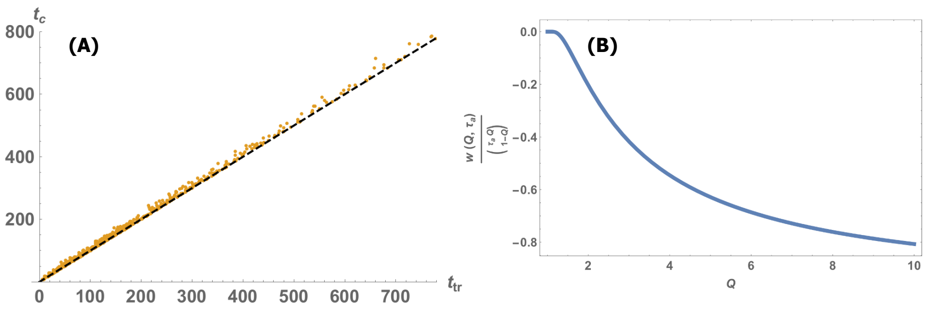

Figure 3: (A) Numerical estimation of the peak position t_c as a function of the transition time t_{tr} calculated from Eq. C.28, for $10 \leq \tau_a \leq 250$ and $1.1 \leq Q \leq 4$. The line $t_c = t_{tr}$ (black, dashed) is also plotted as a guide for the eye. (B) The ratio of the correction term in Eq. C.28 and the scaling argument of the transition time t_r (i.e. - $(\tau_a Q)/(Q-1)$ as a function of Q. For $Q \sim 1$ the correction term is negligible (i.e - the ratio goes to zero), while for $Q \ll 1$ the ratio approaches -1. This implies that as Q increases, the transition time becomes shorter.

long times $t \gg \tau_a$ the pacing is maximal.

In Fig 2 we plot the time evolution of the phase ϕ_p and its time derivative $\dot{\phi}_p$ (both normalized for convenience) as a function of reduced time $t^* = \Delta\omega t$, for various values of the scaled adaptation time $\Delta\omega\tau_a$. One can see that as τ_a increase, the phase relaxes to a constant value at longer and longer times. This means that the time required to achieve synchronization becomes much longer. Also, note that in all cases where the cell is eventually synchronized, there is a slight overshoot (corresponding to a single root for the matching derivative) followed by a smooth relaxation to the steady-state synchronized phase. This peak time marks the transition from oscillatory, to relaxation behavior. We therefore use the peak time t_c as a measure of the time required to synchronize the cell.

In Eq. 2, the transition from oscillatory to relaxation dynamics happens when the parameter Q changes sign. This suggests that for the analogous Eq. C.26, the transition would occur at time t_{tr} for which:

$$\frac{(Q-1)}{Q} = \frac{\tau_a}{t_{tr}}\left(1 - e^{-t_{tr}/\tau_a}\right) \tag{C.27}$$

Eq. C.27 can be solved analytically for all $Q > 1$, which yields:

$$t_{tr} = \frac{\tau_a Q}{Q-1} + w(Q,\tau_a), \qquad w(Q,\tau_a) = \tau_a W\left(\frac{Q}{1-Q}e^{-\frac{Q}{1-Q}}\right) \tag{C.28}$$

where $W(z)$ is the Lambert (Product-Log) function. In Fig. 3A we show the transition time t_c estimated numerically, plotted against the (corrected) transition time t_{tr} evaluated analytically from Eq. C.28, for varying values of Q and τ_a. As predicted by our analysis, numerical solutions of the equation collapse

rather well onto a linear curve of slope 1. In Fig. 3B we plot the ratio of the correction $w(Q, \tau_a)$ and the scaling argument given in the main text (derived neglecting $w(Q, \tau_a)$). For $Q \sim 1$ the correction is negligible (the ratio is 0), showing that the scaling argument is valid. As Q increase, $w(Q, \tau_a)$ becomes more and more negative, effectively shortening the transition time. For $Q \gg 1$ the scaling argument and $w(Q, \tau_a)$ are comparable in size and opposite in sign, leading to a shortened transition time that scales as $\sim \tau_a/Q$.

D Dynamics of stochastic beating

D.1 Coherence of beating for weak pacing

We start by adding stochastic term to Eq. B.11, describing the intrinsic random forces within the cell due to various biological processes:

$$\ddot{\chi} - \rho\dot{\chi} + \lambda\dot{\chi}^3 + \omega_c\chi = A_p\cos(\omega_p t) + \chi_s(t) \tag{D.29}$$

Since we are interested in the long-time coherence of beating, we approximate the short-time noise by a random function $\chi_s(t)$, which is Gaussian noise with mean $\langle\chi_s(t)\rangle = 0$ and temporal correlation $\langle\chi_s(t)\chi_s(t')\rangle \sim (2D^*)\delta(t-t'))$ with D^* a measure for the magnitude of the noise. If the external pacing force is weak ($A_p \ll 1$), and the noise varies on a time-scale longer than a single period, we expect that on average the cell will beat with a frequency ω_c. To avoid the mathematical complexities of multiplicative noise, we follow the approach described by Hanggi & Riseborough, where a rotating frame of reference is adopted [14]:

$$\begin{aligned}\chi =& y_i\cos(\omega_c t) - y_o\sin(\omega_c t)\\ \dot{\chi} =& -\omega_c(y_i\sin(\omega_c t) + y_o\cos(\omega_c t))\end{aligned} \tag{D.30}$$

where y_i and y_o are the in-phase and out-of-phase components of the beating amplitude respectively. Inserting these expressions into Eq. D.29, and averaging over a single cycle as before yields the two dynamical equations for the amplitude components:

$$\begin{aligned}\dot{y}_i =& \frac{y_i}{2}\left(\rho - \frac{3}{4}\lambda\omega_c^2(y_i^2+y_o^2)\right) - \frac{A_p\omega_c}{\pi}\frac{\sin(\pi\frac{\omega_p}{\omega_c})}{(\omega_c^2-\omega_p^2)}\sin(\pi\frac{\omega_p}{\omega_c}) - \frac{\bar{\chi}_{s,i}(t)}{\omega_c}\\ \dot{y}_o =& \frac{y_o}{2}\left(\rho - \frac{3}{4}\lambda\omega_c^2(y_i^2+y_o^2)\right) + \frac{A_p\omega_p}{\pi}\frac{\sin(\pi\frac{\omega_p}{\omega_c})}{(\omega_c^2-\omega_p^2)}\cos(\pi\frac{\omega_p}{\omega_c}) - \frac{\bar{\chi}_{s,o}(t)}{\omega_c}\end{aligned} \tag{D.31}$$

with $\bar{\chi}_{s,i}(t)$ and $\bar{\chi}_{s,o}(t)$ the (time averaged) noise in the in and out of phase components with the property $\langle\bar{\chi}_{s,j}(t)\bar{\chi}_{s,j}(t')\rangle = 2D^*/\omega_c^2\delta(t-t') = 2D'\delta(t-t')$, and $\langle\bar{\chi}_{s,j}(t)\bar{\chi}_{s,k}(t)\rangle = 0$ if $j \neq k$. The two coupled Langevin equations above are, in general, difficult to solve. We therefore go from the Langevin equations, to the Fokker-Planck (FP) formulation for the probability density $P(y_i, y_o, t; y_i^0, y_o^0, 0)$, which is the probability that amplitude components y_i, y_o at time t have certain values, given that at time $t = 0$ those had the values y_i^0, y_o^0. In vector form, the FP equation is derived [15] from probability conservation, and is given by:

$$\dot{P} = -\vec{\nabla}\cdot(\vec{R}P) + D'\ \nabla^2 P \tag{D.32}$$

with $\vec{R}$ the n-dimensional drift term vector, corresponding to the non-stochastic

part of each of the Langevin equations. Accordingly, for Eq. D.31 we have:

$$\begin{aligned} R_i(y_i, y_o) =& \frac{y_i}{2}\left(\rho - \frac{3}{4}\lambda\omega_c^2(y_i^2 + y_o^2)\right) - \frac{A_p\omega_c}{\pi}\frac{\sin(\pi\frac{\omega_p}{\omega_c})}{(\omega_c^2 - \omega_p^2)}\sin(\pi\frac{\omega_p}{\omega_c}) \\ R_o(y_i, y_o) =& \frac{y_o}{2}\left(\rho - \frac{3}{4}\lambda\omega_c^2(y_i^2 + y_o^2)\right) + \frac{A_p\omega_p}{\pi}\frac{\sin(\pi\frac{\omega_p}{\omega_c})}{(\omega_c^2 - \omega_p^2)}\cos(\pi\frac{\omega_p}{\omega_c}) \end{aligned} \tag{D.33}$$

We now express these equations in a polar representation where $y_i = A(t)\cos(\phi_c(t))$, $y_o = A(t)\sin(\phi_c(t))$. The FP equation in this representation is:

$$\dot{P} = -\frac{1}{A}\frac{\partial}{\partial A}(A\ R_a\ P) - \frac{\partial}{\partial\phi_c}(R_\phi P) + D'\left(\frac{1}{A}\frac{\partial}{\partial A}\left(A\frac{\partial P}{\partial A}\right) + \frac{1}{A^2}\frac{\partial^2 P}{\partial\phi_c^2}\right) \tag{D.34}$$

where the drift terms become:

$$\begin{aligned} R_a(A, \phi_c) =& \cos(\phi_c)R_i(A, \phi_c) + \sin(\phi_c)R_o(A, \phi_c) + \frac{D'}{\omega_c A} \\ R_\phi(A, \phi_c) =& -\frac{\sin(\phi_c)}{A}R_i(A, \phi_c) + \frac{\cos(\phi_c)}{A}R_o(A, \phi_c) \end{aligned} \tag{D.35}$$

Inserting the transformation into Eqs. D.33 and D.35 and re-arranging gives:

$$\begin{aligned} R_a(A, \phi_c) =& \frac{A}{2}\left(\rho - \frac{3}{4}\lambda\omega_c^2 A\right) - \alpha^*\sin(\phi_c - \beta) + \frac{D'}{\omega_c A} \\ R_\phi(A, \phi_c) =& \frac{\alpha^*}{A}\cos(\phi_c - \beta) \end{aligned} \tag{D.36}$$

Where we define:

$$\begin{aligned} \alpha^* =& \frac{A_p\omega_c}{\pi}\frac{\sin(\pi\frac{\omega_p}{\omega_c})}{(\omega_c^2 - \omega_p^2)}\sqrt{\sin^2(\pi\frac{\omega_p}{\omega_c}) + \left(\frac{\omega_p}{\omega_p}\right)^2\cos^2(\pi\frac{\omega_p}{\omega_c})} \\ \beta =& \arctan\left(\frac{\omega_c}{\omega_p}\tan(\pi\frac{\omega_p}{\omega_c})\right) \end{aligned} \tag{D.37}$$

Eq. D.34 still contain terms that couple the amplitude A and phase ϕ_c. To further simplify the problem, we consider the case of weak external force $A_p \ll 1$, and examine the limit of weak noise $\sqrt{D'/\omega_c\rho} \ll A$. For this case, the amplitude has small fluctuations around its steady state value $A_s = \sqrt{(4\rho/3\lambda\omega_p^2)}$. Applying these simplifications, and defining $D = D'/A_s^2$ and $\alpha = \alpha^*/A_s$ yields the simplified FP equation described in the main text:

$$\dot{P} = -\frac{\partial}{\partial\phi_c}(\alpha\cos(\phi_c - \beta)P) + D\frac{\partial^2 P}{\partial\phi_c^2} \tag{D.38}$$

with steady-state solution:

$$P_s(\phi) = \frac{\exp\left(\frac{\alpha}{D}\sin(\phi_c - \beta)\right)}{2\pi\ I_0(\alpha/D)} \tag{D.39}$$

We further expand Eq. D.39 around its peak $\phi_c = (\beta + \pi/2)$ to get:

$$P_s(\phi) \approx \frac{e^{\frac{\alpha}{D}\left(1-\frac{1}{2}\left(\phi_c-\beta-\frac{\pi}{2}\right)^2\right)}}{2\pi\ I_0(\alpha/D)} \tag{D.40}$$

which for small noise ($D \to 0$) yields:

$$\lim_{D\to 0} P_s \approx \lim_{D\to 0}\sqrt{\frac{\alpha}{2\pi D}}e^{-\frac{\alpha}{2D}\left(\phi_c-\beta-\frac{\pi}{2}\right)^2} = \delta(\phi_c - (\beta + \pi/2)) \tag{D.41}$$

Thus the steady-state distribution for the case of small noise can be approximated by a delta-function of the phase around its steady state value of $\phi_c = \beta + \pi/2$.

D.2 Dynamics and noise for a strongly paced cell

For a strong external field (pacing), we expect the cell to be entrained to the probe, even in the presence of noise. We therefore use the reference state where the phase $\phi_p(t)$ is relative to $\omega_p t$ as in Sec. B (i.e. - $\chi(t) = A(t)\cos(\omega_p t + \phi_p(t))$). The appropriate FP equation (derived by Hanggi & Riseborough [14] is:

$$\dot{P} = -\frac{\partial}{\partial \phi_p}\left((\Delta\omega + \alpha\sin(\phi_p))P\right) + D\frac{\partial^2 P}{\partial \phi_p^2} \tag{D.42}$$

For $\alpha \sim A_p/A_s$ a measure of the external pacing strength. For a relatively large amplitude of external force, the steady state probability density has been calculated by Hanggi & Riseborough [14]:

$$P_s(\phi_p) = \frac{1}{Z}e^{-V(\phi_p)/D}\int_{\phi_p}^{\phi_p+2\pi} e^{V(\phi')/D}\ d\ \phi' \tag{D.43}$$

with Z the normalization and $V(\phi_p) = -(\Delta\omega\phi_p - \alpha\cos(\phi_p))$ a linearly-biased, periodic potential due to the probe. Eq.D.43 can be further simplified by defining the transformation $\psi = \phi' - \phi_p$. Writing P_s in terms of ϕ_p and ψ we have:

$$\begin{aligned} P_s(\phi_p) &= \frac{1}{Z}A(\phi_p)\int_0^{2\pi} B(\phi_p, \psi)\ d\ \psi \\ A(\phi_p) &= e^{-\frac{\alpha}{D}\cos(\phi_p)} \\ B(\phi_p, \psi) &= e^{-\frac{\Delta\omega}{D}\psi + \frac{\alpha}{D}\cos(\psi+\phi_p)} \end{aligned} \tag{D.44}$$

To proceed, we define the following expansions in terms of the modified Bessel function $I_n(z)$ [16]:

$$
\begin{aligned}
e^{a\cos(b)} =& I_0(a) + 2\sum_{n=1}^{\infty}\cos(nb) I_n(a) \\
e^{-a\cos(b)} =& I_0(a) + 2\sum_{n=1}^{\infty}(-1)^n\cos(nb) I_n(a)
\end{aligned}
\tag{D.45}
$$

using the definition of the normalization factor

$$
Z = \int_0^{2\pi} A(\phi_p)\ d\phi \int_0^{2\pi} B(\phi_p, \psi)\ d\psi \tag{D.46}
$$

we calculate the integrals in Eq. D.44,D.46 analytically [16]. This yields the steady-state probability distribution:

$$
P_s(\phi_p) = \frac{e^{-\frac{\alpha}{D}\cos(\phi_p)}\left(\left(\frac{D}{\Delta\omega}\right)^2 I_0\left(\frac{\alpha}{D}\right) + 2\sum_{n=1}^{\infty}\left(\frac{\cos(n\phi_p)-\left(\frac{Dn}{\Delta\omega}\right)\sin(n\phi_p)}{n^2+\left(\frac{D}{\Delta\omega}\right)^2}\right) I_n\left(\frac{\alpha}{D}\right)\right)}{2\pi\left(\left(\frac{D}{\Delta\omega}\right)^2 I_0\left(\frac{\alpha}{D}\right)^2 + 2\sum_{m=1}^{\infty}\frac{I_m\left(\frac{\alpha}{D}\right)^2}{m^2+\left(\frac{D}{\Delta\omega}\right)^2}\right)} \tag{D.47}
$$

In Fig. 4 we plot Eq. D.47 for various values of the scaled amplitude of probe α/D and detuning $\Delta\omega/D$. The probability distribution has the same general characteristics as the stationary probability distribution for the phase of a weakly paced cell presented in Eq. D.39. The distribution is slightly asymmetric about its peak, and this asymmetry is increasing as the detuning $\Delta\omega$ increases, or as the forcing α decreases. The peak of the distribution shifts towards lower values of ϕ_p as both α and $\Delta\omega$ increase. For very weak noise, the zero order term in Eq. D.47 is negligible, and one can expand all the modified bessel functions in $D \ll 1$, and around $\phi_p = \pi$, to yield:

$$
\begin{aligned}
\lim_{D\to 0} P_s &\approx \frac{e^{-\frac{\alpha}{D}\cos(\phi_p)}(2\pi\alpha/D)^{-1/2}e^{-\frac{\alpha}{D}}\sum_n \frac{1}{n^2}}{2\pi(2\pi\alpha/D)^{-1}e^{-2\frac{\alpha}{D}}\sum_m \frac{1}{m^2}} \\
&= \sqrt{\frac{\alpha}{2\pi D}}e^{-\frac{\alpha}{D}(\phi_p-\pi)^2}
\end{aligned}
\tag{D.48}
$$

which is again the definition of a delta-function $\delta(\phi_p - \pi)$. The shift of the peak from $\phi_p = (\beta - \pi/2)$ is a result of our shift of the frame of reference from ϕ_c to ϕ_p.

This similarity between the steady-state probability densities allows us to examine the evolution in time of the probability density $P(\phi, t)$ by evaluating the moments of the distribution. To this end, we modify the method described by Saito [17], by assuming that the time-dependent probability distribution that

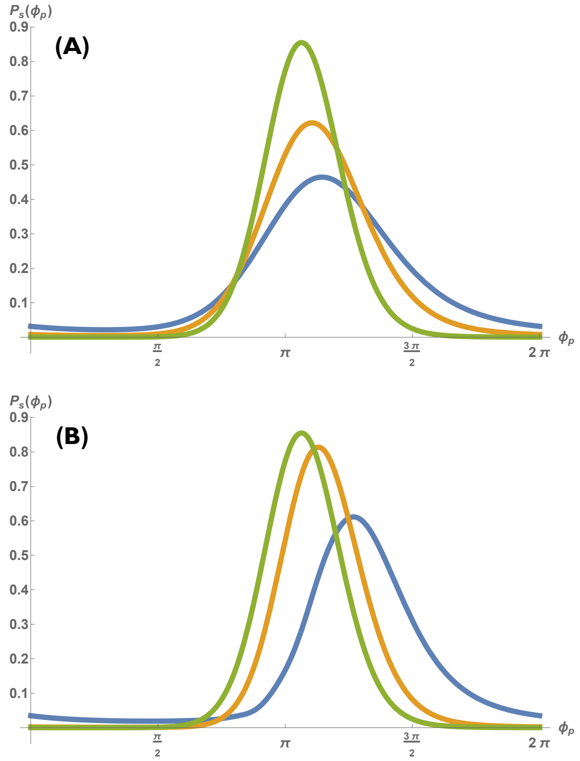

Figure 4: Stationary probability distribution $P_s(\phi_p)$ for the analytic expression in Eq. D.47, taken up to the 10th term in the sum $(1 < n, m < 10)$. (A) Fixed detuning $\Delta\omega/D = 1$ and increasing forcing $f_s/D = 2$ (blue), $f_s/D = 3$(orange) and $f_s/D = 5$, (green). (B) Fixed forcing $f_s/D = 5$ and decreasing detuning $\Delta\omega/D = 4$ (blue), $\Delta\omega/D = 2$ (orange) and $\Delta\omega/D = 1$ (green).

satisfies Eq. D.42 has a general form similar to the steady-state distribution of Eq. D.39, but with time dependent coefficients:

$$P(\phi, t) = \frac{\exp\left(\nu(t)\cos(\phi_s - \mu(t))\right)}{2\pi\ I_0(\nu(t)} \tag{D.49}$$

where $\mu(t)$ and $\nu(t)$ are the two time dependent variables related to peak and width of the probability distribution. By introducing Eq. D.49 into Eq. D.42, and integrating over the phase, we get two dynamical equations for the peak and width:

$$\begin{aligned}\dot{\mu} &= \Delta\omega - f_s \cos(\mu)\left(\frac{I_0(\nu)}{I_1(\nu)} - \frac{1}{\nu}\right) \\ \dot{\nu} &= -\frac{\left(D + f_s \frac{\sin(\mu)}{\nu}\right)}{2\left(\frac{I_0(\nu)}{I_1(\nu)} - \frac{I_1(\nu)}{I_0(\nu)} - \frac{1}{\nu}\right)}\end{aligned} \tag{D.50}$$

For small noise, this set of equations yields the steady state expressions:

$$\begin{aligned}\mu_s &\approx \arccos(\Delta\omega/f_s) \\ \nu_s &\approx \frac{f_s}{D}\sqrt{1 - \left(\frac{\Delta\omega}{f_s}\right)^2}\end{aligned} \tag{D.51}$$

which, for a strongly paced cell ($f_s \gg \Delta\omega$), is identical to the result in Eq. D.39. This implies that the steady state distribution is mainly determined by the ratio of the reduced amplitude of the external force f_s to the noise D.

In the limit of small noise, μ approaches its steady-state value in Eq. D.51 on short time scales of order $\sim 1/f_s$, and the two dynamical equations in D.50 are decoupled. By expanding For $D \ll 1$ we can find the approximate expression for the width:

$$\nu(t) = \nu_s \frac{e^{f_s t}}{e^{f_s t} - 1} \tag{D.52}$$

This expression is used to estimate the correlation in displacement:

$$\langle x(\phi, t)x(\phi_0, 0)\rangle = \int_0^{2\pi} x_0 x(\phi, t)P(\phi, t)\, d\phi\ \sim\ \cos(\omega_p t + \mu_s)\left(1 - \frac{D}{\nu_s}\left(1 - e^{-f_s t}\right)\right) \tag{D.53}$$

where x_0, ϕ_0 are arbitrary initial displacement and phase respectively. Note that at long times the correlation function decays to steady state, oscillatory behavior with frequency ω_p, and an amplitude smaller than the deterministic case by a term proportional to the noise, D. This implies that as long as the cell is paced by an external force, the phase does not become decorrelated in time, and shows only small fluctuations about its steady-state, oscillatory solution.

References

[1] Sprott, J. C. *Elegant chaos: Algebraically Simple Chaotic Flows.* (World Scientific, 2010).

[2] Jülicher, F. & Prost, J. Spontaneous oscillations of collective molecular motors. *Phys. Rev. Lett.*, **78(23),** 4510, (1997).

[3] Jülicher, F. Mechanical oscillations at the cellular scale. *Cr. Acad. Sci. IV-Phys.*, **2(6),** 849–860, (2001).

[4] Bershadsky, A. D., Balaban, N. Q. & Geiger, B. Adhesion-dependent cell mechanosensitivity. *Annu. Rev. Cell Dev. B.*, **19(1),** 677–695, (2003).

[5] Peter, A. K., Cheng, H., Ross, R. S., Knowlton, K. U. & Chen, J. The costamere bridges sarcomeres to the sarcolemma in striated muscle. *Prog. Pediatr. Cardiol.*, **31(2),** 83–88, (2011).

[6] Bers, D. M. Cardiac excitation–contraction coupling. *Nature*, **415(6868),** 198–205, (2002).

[7] Eisner, D.A., Choi, H. S., Diaz, M. E., Oneill, S. C. & Trafford, A. W. Integrative analysis of calcium cycling in cardiac muscle. *Circ. Res.*, **87(12),** 1087–1094, (2000).

[8] Reed, A., Kohl, P. & Peyronnet, R. Molecular candidates for cardiac stretch-activated ion channels. *Glob. Cardiol. Sci. Pract.*, **2014(2),** (2014).

[9] Schwarz, U. S. & Safran, S A. Physics of adherent cells. *Rev. Mod. Phys.*, **85(3),** 1327-1381, (2013).

[10] Purcell, E. M. Life at low reynolds number. *Am. J. Phys.*, **45(1),** 3–11, (1977).

[11] Stam, S., Alberts, J.,Gardel, L.M. & Munro, E. Force generation by Myosin II filaments in compliant networks. *arXiv*, **arXiv:1404.3262**, (2014).

[12] Verhulst, F. Nonlinear differential equations and dynamical systems. *Springer Science & Business Media*, (2006).

[13] Nitsan, I., Drori, S., Lewis, Y. E., Cohen, S. & Tzlil, S. Mechanical communication in cardiac cell synchronized beating. *Nat. Phys.*, (2016).

[14] Hanggi, P. & Riseborough, P. Dynamics of nonlinear dissipative oscillators. *Am. J. Phys.*, **51(4),** 347–352, (1983).

[15] Risken, H. *Fokker-Planck Equation.* (Springer, 1984).

[16] Arenas, A. & Vicente, C. J. P. Exact long-time behavior of a network of phase oscillators under random fields. *Phys. Rev. E*, **50(2),** 949, (1994).

[17] Saito, Y. Self-consistent calculation of statics and dynamics of the roughening transition. *Z. Phys. B Con. Mat.*, **32(1),** 75–82, (1978).
